# A randomized, controlled study of peginterferon lambda-1a/ribavirin ± daclatasvir for hepatitis C virus genotype 2 or 3

**DOI:** 10.1186/s40064-016-2920-z

**Published:** 2016-08-19

**Authors:** Graham R. Foster, Kazuaki Chayama, Wan-Long Chuang, Hugo Fainboim, Martti Farkkila, Adrian Gadano, Giovanni B. Gaeta, Christophe Hézode, Yukiko Inada, Jeong Heo, Hiromitsu Kumada, Sheng-Nan Lu, Patrick Marcellin, Christophe Moreno, Stuart K. Roberts, Simone I. Strasser, Alexander J. Thompson, Joji Toyota, Seung Woon Paik, John M. Vierling, Anna L. Zignego, David Cohen, Fiona McPhee, Megan Wind-Rotolo, Subasree Srinivasan, Matthew Hruska, Heather Myler, Simon D. Portsmouth

**Affiliations:** 1 Department of Hepatology, Queen Mary University of London, 4 Newark Street, London, UK; 2 Department of Gastroenterology and Nutrition, Hiroshima University, 1-2-3, Kasumi, Minami-ku, Hiroshima-shi, Hiroshima, Japan; 3 Hepatobiliary Division, Department of Internal Medicine, Kaohsiung Medical University Hospital, Kaohsiung Medical University, No. 100, Shih-Chuan 1st Road, Kaohsiung, Taiwan; 4 Liver Unit, Hospital F. J. Muñiz, Uspallata 2272, Buenos Aires, Argentina; 5 Helsinki University and Clinic of Gastroenterology, University of Helsinki, Haartmaninkatu 4, Helsinki, Finland; 6 Liver Unit, Hospital Italiano de Buenos Aires, Buenos Aires, Argentina; 7 Internal and Specialistic Medicine, Viral Hepatitis Unit, Second University of Naples, Via Pansini 5 Bld.3, 08131 Naples, Italy; 8 Hepatology, Henri Mondor Hospital, AP-HP, INSERM U955, University Paris-Est, 51 av du Maréchal de Lattre de Tassigny, Créteil, France; 9 Center for Digestive and Liver Diseases, Miyazaki Medical Center Hospital, 2-16 Takamtsu-cho, Miyazaki-shi, Miyazaki, Japan; 10 Department of Internal Medicine, Pusan National University and Medical Research Institute, College of Medicine, Pusan National University Hospital, 179 Gudeok-Ro, Seo-gu, Pusan, Republic of Korea; 11 Department of Hepatology, Toranomon Hospital, 2-2-2 Toranomon, Minato-ku, Tokyo, Japan; 12 Section of Hepatology, Division of Hepatogastroenterology, Department of Internal Medicine, Kaohsiung Chang Gung Memorial Hospital and Chang Gung University College of Medicine, 123 Taoei Road, Niaosung District, Kaohsiung, 833 Taiwan; 13 Hepatology, Hôpital Beaujon, 100 Bd du Général Leclerc, 92110 Clichy, France; 14 Department of Gastroenterology, Hepatopancreatology and Digestive Oncology, CUB Hôpital Erasme, Université Libre de Bruxelles, 808 Route de Lennik, 1070 Brussels, Belgium; 15 Gastroenterology, Alfred Hospital, 99 Commercial Road, Melbourne, Australia; 16 Department of Medicine, Monash University, Melbourne, Australia; 17 AW Morrow Gastroenterology and Liver Centre, Royal Prince Alfred Hospital, Missenden Rd, Camperdown, Sydney, NSW 20150 Australia; 18 Department of Gastroenterology, St. Vincent’s Hospital and the University of Melbourne, SVHM Level 4 Daly Wing, 35 Victoria Pde, PO Box 29000, Fitzroy, Australia; 19 Department of Hepatology, Sapporo Kosei General Hospital, Kita 3 Higashi 8-5, Chuo-ku, Sapporo, Japan; 20 Department of Medicine, Samsung Medical Centre, Sungkyunkwan University School of Medicine, 81 Irwon-ro, Gangnam-gu, Seoul, Republic of Korea; 21 Baylor Liver Health, Baylor College of Medicine, 6620 Main Street, Suite 1425, Houston, TX USA; 22 Department of Experimental and Clinical Medicine, MASVE Center, Universita Di Firenze, Largo Brambilia, 3, 50134 Florence, Italy; 23 Bristol-Myers Squibb Global Biometric Sciences, 5 Research Parkway, Wallingford, CT USA; 24 Discovery Virology, Bristol-Myers Squibb, 5 Research Parkway, Wallingford, CT USA; 25 Exploratory Clinical and Translational Research, Bristol-Myers Squibb Company, Princeton, NJ USA; 26 Global Clinical Research, Bristol-Myers Squibb Company, 5 Research Parkway, Wallingford, CT 06492 USA; 27 Clinical Pharmacology and Pharmacometrics, Bristol-Myers Squibb Company, Hopewell, NJ USA; 28 Analytical and Bioanalytical Development, Bristol Myers Squibb Company, Princeton, NJ USA; 29 Shionogi Inc., 300 Campus Drive, Florham Park, NJ 07932 USA

**Keywords:** Hepatitis C virus, Genotype 2, Genotype 3, Peginterferon lambda-1a, Peginterferon alfa-2a

## Abstract

**Background and purpose:**

Peginterferon Lambda was being developed as an alternative to alfa interferon for the treatment of chronic hepatitis C virus (HCV) infection. We compared peginterferon Lambda-1a plus ribavirin (Lambda/RBV) and Lambda/RBV plus daclatasvir (DCV; pangenotypic NS5A inhibitor) with peginterferon alfa-2a plus RBV (alfa/RBV) in treatment-naive patients with HCV genotype 2 or 3 infection.

**Methods:**

In this multicenter, double-blind, phase 3 randomized controlled trial, patients were assigned 2:2:1 to receive 24 weeks of Lambda/RBV, 12 weeks of Lambda/RBV + DCV, or 24 weeks of alfa/RBV. The primary outcome measure was sustained virologic response at post-treatment Week 12 (SVR12).

**Results:**

Overall, 874 patients were treated: Lambda/RBV, *n* = 353; Lambda/RBV + DCV, *n* = 349; alfa/RBV, *n* = 172. Patients were 65 % white and 33 % Asian, 57 % male, with a mean age of 47 years; 52 % were infected with genotype 2 (6 % cirrhotic) and 48 % with genotype 3 (9 % cirrhotic). In the Lambda/RBV + DCV group, 83 % (95 % confidence interval [CI] 78.5, 86.5) achieved SVR12 (90 % genotype 2, 75 % genotype 3) whereas SVR12 was achieved by 68 % (95 % CI 63.1, 72.9) with Lambda/RBV (72 % genotype 2, 64 % genotype 3) and 73 % (95 % CI 66.6, 79.9) with peginterferon alfa/RBV (74 % genotype 2, 73 % genotype 3). Lambda/RBV + DCV was associated with lower incidences of flu-like symptoms, hematological abnormalities, and discontinuations due to adverse events compared with alfa/RBV.

**Conclusion:**

The 12-week regimen of Lambda/RBV + DCV was superior to peginterferon alfa/RBV in the combined population of treatment-naive patients with genotype 2 or 3 infection, with an improved tolerability and safety profile compared with alfa/RBV.

**Electronic supplementary material:**

The online version of this article (doi:10.1186/s40064-016-2920-z) contains supplementary material, which is available to authorized users.

## Background

Chronic hepatitis C virus (HCV) infection affects up to 170 million people worldwide based on serologic data (Lavanchy 2009), resulting in approximately 500,000 deaths each year (World Health Organization 2012). HCV comprises 7 major genotypes and 67 subtypes; genotypes 1, 2, and 3 are the most widely distributed and the most studied therapeutically (Smith et al. 2014). Genotype 2 is prevalent in South America and Asia, whereas genotype 3 is common in Europe (European Association for the Study of the Liver 2014), the United States, Australia, and southern Asia (Ansaldi et al. 2014).

Previously, HCV genotype 2 or 3 infection was treated primarily with 24 weeks of peginterferon alfa-2a plus ribavirin (alfa/RBV). Alfa/RBV therapy is subject to a number of limitations; among these are frequent, sometimes treatment-limiting adverse events (AEs) including hemolytic anemia and other cytopenias (Sulkowski et al. 2011). Genotype 3 infection has been associated with poorer outcomes than genotype 2, including a higher incidence of steatosis (Matos et al. 2006), accelerated fibrosis (Bochud et al. 2009; Probst et al. 2011), increased risk of hepatocellular cancer, and lower sustained virologic response (SVR) rates following treatment with alfa/RBV or oral direct-acting antiviral (DAA)-based regimens (Zeuzem et al. 2004; Andriulli et al. 2008).

Oral DAA regimens are now replacing interferon-based treatment for chronic HCV infection; however, DAA options for genotypes 2 and 3 are more restricted than those for genotype 1. Currently approved regimens for genotype 2-infected patients include sofosbuvir (SOF) plus RBV and peginterferon plus RBV in the US and EU, and SOF plus velpatasvir (VEL) in the US. Approved regimens for genotype 3-infected patients include combinations of SOF plus RBV, SOF plus daclatasvir (DCV) with or without RBV, SOF plus peginterferon and RBV, and peginterferon plus RBV; in addition, SOF plus VEL was recently approved in the US (Bristol Myers Squbb Pharmaceuticals Ltd 2014; Bristol-Myers Squibb 2016). DCV + SOF with or without RBV is a preferred option for treating HCV genotype 3-infected patients according to guidelines issued by the American Association for the Study of Liver Disease and the European Association for the Study of the Liver (European Association for the Study of the Liver 2014; AASLD/IDSA HCV Guidance Panel 2015).

Peginterferon lambda-1a (Lambda) is a pegylated Type III interferon with HCV antiviral activity similar to that of alfa interferons; however, Lambda utilizes a unique receptor with more restricted tissue distribution than the alfa receptor (Andersen et al. 2013). In the phase 2 EMERGE study, similar SVR rates were achieved with Lambda/RBV and alfa/RBV in previously untreated genotype 1–4 infection. However, Lambda/RBV exhibited improved tolerability characterized by fewer musculoskeletal and influenza-like events (Muir et al. 2014) and a better hematologic profile, including amelioration of RBV-associated anemia through compensatory erythropoiesis (Everson et al. 2011).

Daclatasvir (DCV) is an NS5A replication complex inhibitor DAA with in vitro activity and clinical data against HCV genotypes 1–6 (Hézode et al. 2016; Welzel et al. 2016; Sulkowski et al. 2014; Gao 2013; Poordad et al. 2016; Nelson et al. 2015; Wyles et al. 2015). DCV has been approved in the US, Europe, Japan, and multiple countries across the Americas, Middle East, and Asia Pacific region. We report the results of a phase 3 study of Lambda/RBV, with and without DCV, versus alfa/RBV in previously untreated patients with genotype 2 or 3 infection.

## Methods

### Study design

This randomized, double-blind, multinational phase 3 study (ClinicalTrials.gov identifier NCT01616524) enrolled treatment-naive patients with genotype 2 or 3 infection from 124 clinical centers in 18 countries in Europe (Belgium, Finland, France, Greece, Italy, Netherlands, United Kingdom), Asia (Japan, Korea, Singapore, Hong Kong, Taiwan), and South America (Argentina, Mexico), plus the USA, Russia, Australia, and New Zealand between 20 July 2012 and 15 August 2013. Eligible patients were randomly assigned 2:2:1 to treatment with either (a) 24 weeks of Lambda/RBV (first 12 weeks with a DCV placebo); (b) 12 weeks of Lambda/RBV plus DCV 60 mg once daily, or (c) 24 weeks of alfa/RBV (first 12 weeks with a DCV placebo). Patients receiving 24 or 12 weeks of therapy were followed up for 24 or 48 weeks post-treatment, respectively. Both peginterferons were self-administered at a dose of 180 μg once weekly by subcutaneous injection. RBV 400 mg was taken orally twice daily with food. DCV 60 mg was taken orally once daily, with or without food.

Randomization was via an interactive voice response system designated by the study sponsor. Randomization was stratified by baseline HCV RNA (<800,000 IU/mL or ≥800,000 IU/mL), cirrhosis status, region (Japan vs the rest of the world), and HCV genotype. Study enrolment was capped by genotype (neither genotype 2 nor 3 could comprise more than 60 % of total enrollment), cirrhosis status (no more than 20 % of patients with compensated cirrhosis in any treatment arm), and region (Japanese patients limited to approximately 70 overall).

### Ethics, consent and permissions

This study was conducted in accordance with Good Clinical Practice guidelines and in accordance with the ethical principles originating in the Declaration of Helsinki. All patients provided written informed consent. The study protocol and all relevant documents were approved by the appropriate independent ethics committee or institutional review board for each participating site prior to initiation.

### Patients

Eligible patients were men and women aged ≥18 years with a body mass index between 18 and 35 kg/m^2^ at screening. HCV RNA levels were required to be ≥100,000 IU/mL, based on reports of lower SVR rates with alfa/RBV therapy in patients with high viral load, particularly those with genotype 3 infection, suggesting a greater medical need for improved therapies for this patient population (Andriulli et al. 2008; Mecenate et al. 2010). Cirrhosis status was determined by liver biopsy or FibroScan. For liver biopsies, absence of cirrhosis (Ishak fibrosis score ≤4 or Metavir score ≤3) was documented within 3 years prior to study enrollment, while biopsies demonstrating cirrhosis (Ishak score ≥5 or Metavir score = 4) could be from any time prior to enrollment. FibroScan results (cirrhosis defined as ≥14.6 kPa) were required within 1 year of enrollment. Women of child-bearing potential and men with female partners of child-bearing potential were required to use highly effective contraception throughout treatment. Major exclusion criteria included coinfection with hepatitis B virus or human immunodeficiency virus, or a history of hepatocellular carcinoma, decompensated liver disease, or any chronic liver disease other than HCV. Laboratory exclusion criteria included hemoglobin ≤12 g/dL for women and ≤13 g/dL for men, platelets <90 × 10^6^/L, absolute neutrophil count ≤1.5 × 10^9^/L, albumin ≤35 g/L, creatinine clearance ≤50 mL/min, total bilirubin ≥2 mg/dL or >1.8 × the upper limit of normal [ULN for cirrhotics (unless Gilbert’s disease was present)], alanine aminotransferase (ALT) ≥5 × ULN, and electrocardiographic abnormality (QTcF >500 ms).

### Endpoints and assessments

The primary endpoint was sustained virologic response at post-treatment Week 12 (SVR12) in the combined genotype 2 and 3 patient population, defined as a plasma HCV RNA measurement below the assay lower limit of quantitation (LLOQ; 25 IU/mL) target detected or not detected. Secondary efficacy endpoints included the proportion of patients with undetectable HCV RNA at Week 4 (rapid virologic response; RVR), Weeks 4 and 12 (extended rapid virologic response on treatment; eRVR), Week 12 (complete early virologic response; cEVR), end-of-treatment response (EOTR), and the proportion of genotype 3-infected patients with SVR12.

Virologic failure was defined as either on-treatment virologic breakthrough or post-treatment relapse. Breakthrough was defined as a confirmed increase in HCV RNA >1 log_10_ IU/mL above nadir or a confirmed HCV RNA level ≥LLOQ after being <LLOQ. Relapse was defined as HCV RNA ≥LLOQ post-treatment following an EOTR. Virologic futility criteria for early discontinuation of study drug were failure to achieve ≥2 log_10_ IU/mL reduction from baseline at treatment Week 12 with either peginterferon without DCV, and virologic breakthrough in patients receiving Lambda/RBV with DCV.

Safety endpoints included proportions of patients with AEs, serious AEs, dose reductions and discontinuations for AEs, proportions of patients with treatment-emergent cytopenic abnormalities (hemoglobin <10 g/dL, absolute neutrophils <0.75 × 10^9^/L, platelets <50 × 10^9^/L), proportions of patients with on-treatment interferon-related flu-like symptoms (pyrexia, chills, pain), musculoskeletal symptoms (myalgia, arthralgia, back pain), or constitutional symptoms (fatigue, asthenia), and treatment-emergent laboratory abnormalities.

HCV genotype was assessed using the VERSANT HCV genotype 2.0 assay (LiPA; Siemens, Washington, DC, USA) and HCV RNA using the COBAS^®^ AmpliPrep/COBAS^®^ TaqMan^®^ HCV Test version 2.0 (Roche, Pleasanton, CA, USA). HCV RNA measurements were taken at screening, baseline, on-treatment Weeks 1, 2, 4, 8, 12, 16, 20, and 24 (as applicable), and post-treatment Weeks 4, 12, 24, 36, and 48 (as applicable). Population-based sequencing of the HCV NS5A region derived from plasma samples from all patients at baseline, and from patients experiencing virologic failure was performed when HCV RNA was ≥1000 IU/mL.

### Statistical analysis

Efficacy analyses were based on a modified intention-to-treat approach (mITT) including all patients who received at least one dose of study medication. Observed values based on all patients with HCV RNA data at the relevant time point were also derived. Treatment comparisons were conducted for each of the two Lambda-containing arms versus the alfa/RBV arm, each at a significance level of 0.025. For the primary endpoint of SVR12 in the combined genotype 2 and genotype 3 patient population, treatment comparisons of both the Lambda arms versus the alfa arm were conducted as two-stage evaluations where superiority was tested only if non-inferiority was first established. The treatment difference in SVR12 and the associated two-sided 97.5 % confidence interval (CI) were estimated using a Mantel–Haenszel approach stratified by the randomization strata. Non-inferiority was inferred if the lower bound of the 97.5 % CI for the treatment difference exceeded −10 %. Superiority was inferred subsequently if the comparison showed non-inferiority and the lower bound of the 97.5 % CI exceeded 0 %. Because superiority was not evaluated unless non-inferiority was established, no adjustment to the significance level was made.

Target enrollment was 875 patients, randomly assigned in a 2:2:1 distribution of 350 in each Lambda arm and 175 in the alfa/RBV comparator arm. Assuming a −10 % non-inferiority margin and response rates of 80 % for alfa/RBV and 82 % for both Lambda/RBV and Lambda/RBV + DCV, these sample sizes were predicted to give 90 % power to demonstrate the non-inferiority of Lambda to alfa for each comparison (SVR12). Assuming an 80 % response on alfa/RBV and a 92 % response in each Lambda arm, and also assuming a two-sided type I error of 0.025, the above sample sizes were also predicted to give 90 % power to demonstrate superiority of Lambda to alfa for each comparison. A multivariate logistic regression analysis was performed to evaluate the effects of baseline factors on SVR12 rates in patients treated with the DCV-containing regimen. In the logistic regression, the dependent variable was SVR12 status (yes or no) and the independent variables (covariates) were the baseline factors. The probability of achieving SVR12 based on those covariates was estimated using a logistic function, which is the cumulative logistic distribution.

## Results

### Patient disposition and baseline characteristics

**Fig. 1:**
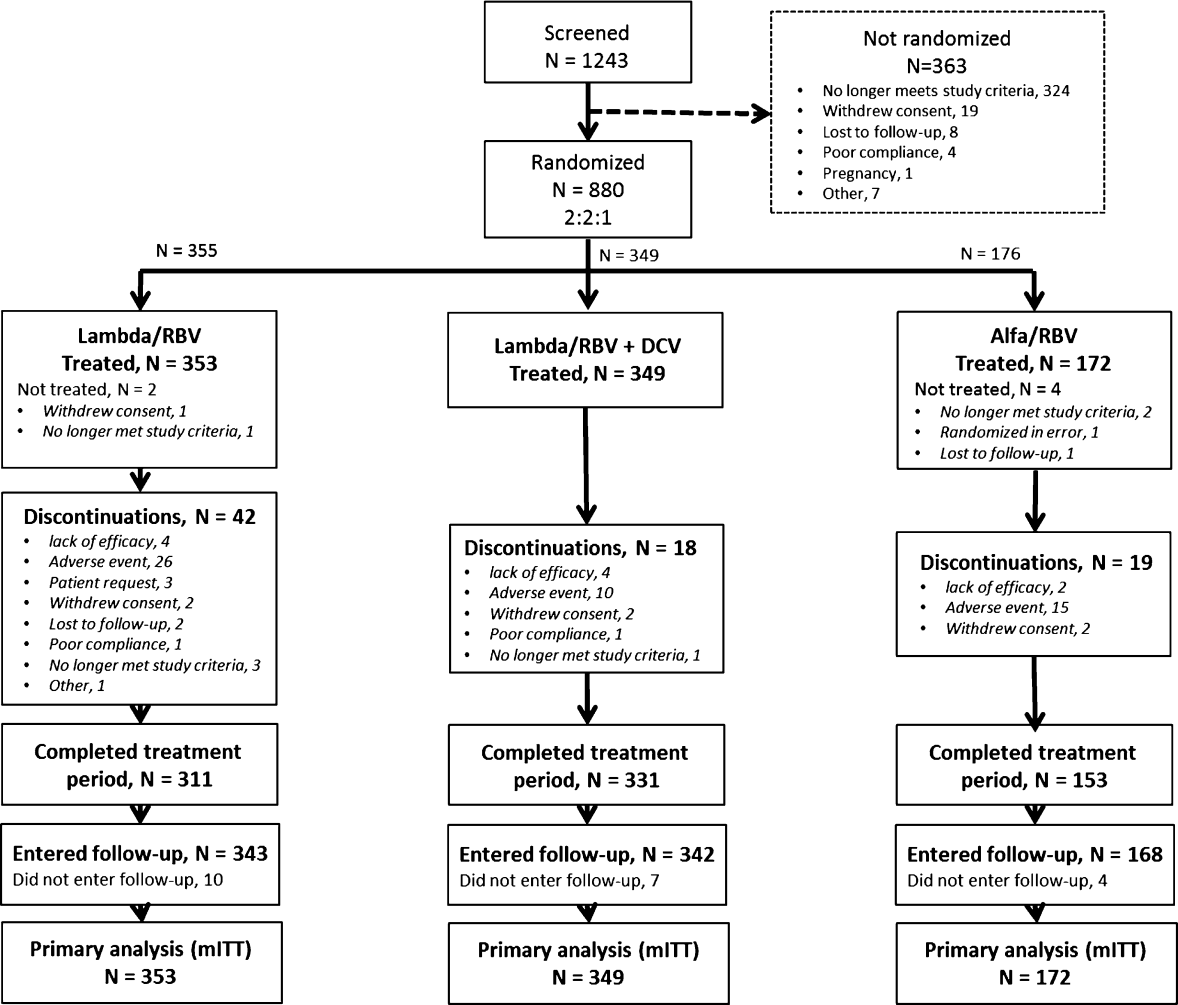
Patient disposition. *Alfa* peginterferon alfa-2a, *DCV* daclatasvir, *Lambda* peginterferon Lambda-1a, *mITT* modified intention-to-treat, *RBV* ribavirin

A total of 1243 patients were screened; 880 were randomized and 874 treated (Fig. 1). Most of the patients screened but not randomized (324/363; 89 %) did not meet study inclusion criteria. Six patients were randomized but not treated; one withdrew consent, one was lost to follow-up, one was a screening failure randomized in error, and three were found not to meet study criteria for drug/alcohol use or other significant protocol deviations.

Cirrhotic patients comprised 7 % (65/874) of the total population (Table 1), with 77 % of diagnoses (50/65) made using FibroScan. Baseline characteristics were generally balanced between treatment arms and between HCV genotype groups, although there was a slightly higher incidence of cirrhosis among patients with genotype 3 (9 %) than genotype 2 (6 %), and a smaller proportion of patients with genotype 3 (46 %) than genotype 2 (60 %) had an *IL28B* CC genotype (rs12979860).

**Table 1:** Baseline demographics and disease characteristics

Parameter	24 weeks lambda/RBV *N* = 353	12 weeks lambda/RBV + DCV *N* = 349	24 weeks alfa/RBV *N* = 172
Age, mean (range), years	48 (20, 72)	47 (18, 74)	46 (21, 73)
Male, *n* (%)	210 (59)	195 (56)	93 (54)
Race/ethnicity, *n* (%)			
White	232 (66)	226 (65)	111 (64)
Black/African American	4 (1)	5 (1)	3 (2)
Asian	113 (32)	118 (34)	55 (32)
Other	4 (1)	0	3 (2)
Hispanic/Latino ethnicity	45 (13)	48 (14)	17 (10)
BL HCV RNA, mean (SD) (log_10_ IU/mL)	6 (1)	6 (1)	6 (1)
HCV RNA distribution (IU/mL)			
≥800,000	269 (76)	276 (79)	132 (77)
HCV genotype, *n* (%)			
Genotype 2	183 (52)	184 (53)	91 (53)
Genotype 3	170 (48)	165 (47)	81 (47)
Cirrhosis, *n* (%)	25 (7)	26 (7)	14 (8)
By biopsy	9 (3)	4 (1)	2 (1)
By FibroScan	16 (5)	22 (6)	12 (7)
*IL28B* genotype (RS12979860), *n* (%)			
CC	180 (51)	192 (55)	94 (55)
CT	145 (42)	126 (36)	68 (40)
TT	27 (8)	28 (8)	10 (6)
Body mass index >30 kg/m^2^, *n* (%)	54 (15)	62 (18)	21 (12)

*alfa* peginterferon alfa-2a, *BL* baseline, *DCV* daclatasvir, *HCV* hepatitis C virus, *Lambda* peginterferon Lambda-1a, *RBV* ribavirin, *SD* standard deviation

### Efficacy

SVR12 was achieved by 83 % (95 % CI 78.5, 86.5) in the Lambda/RBV + DCV group, 68 % (95 % CI 63.1, 72.9) in the Lambda/RBV group, and 73 % (95 % CI 66.6, 79.9) in the alfa/RBV group. In the primary analysis, Lambda/RBV + DCV demonstrated superiority to alfa/RBV, with a treatment difference of 9 % and a 97.5 % CI (0.3, 17.6). In contrast, SVR12 for Lambda/RBV versus alfa/RBV did not meet prespecified non-inferiority criteria, with a treatment difference of −6 % and a 97.5 % CI (−14.9, 3.4) whose lower limit was not above −10 % (Table 2). SVR12 rates were lower in patients with cirrhosis than in those without cirrhosis in all three treatment arms. SVR12 rates in patients with cirrhosis versus those without cirrhosis were 48 % versus 69 % for Lambda/RBV, 65 % versus 84 % for Lambda/RBV + DCV, and 57 % versus 74 % for alfa/RBV.

**Table 2:** Primary and secondary efficacy endpoints

Parameter, % (n/N), except where indicated	24 weeks lambda/RBV *N* = 353	12 weeks lambda/RBV + DCV *N* = 349	24 weeks alfa/RBV *N* = 172
SVR12^a^	68 (240/353)	83 (288/349)	73 (126/172)
Treatment diff. (97.5 % CI)^a^	−6 (−14.9, 3.4)	9 (0.3, 17.6)	N/A
SVR12 genotype 3	64 (109/170)	75 (123/165)	73 (59/81)
Treatment diff. (97.5 % CI)^a^	−9 (−22.3, 4.2)	1 (−11.5, 14.3)	N/A
SVR12 genotype 3 with cirrhosis	29 (4/14)	43 (6/14)	50 (4/8)
SVR12 genotype 2	72 (131/183)	90 (165/184)	74 (67/91)
Treatment diff. (97.5 % CI)	−3 (−15.5, 9.9)	16 (4.2, 27.2)	N/A
SVR12 genotype 2 with cirrhosis	73 (8/11)	92 (11/12)	67 (4/6)
SVR24^b^	66 (232/353)	82 (285/349)	72 (123/172)
Treatment diff. (97.5 % CI)^a^	−6 (−15.5, 2.9)	10 (1.1, 18.4)	N/A
RVR	64 (227/353)	86 (299/349)	58 (99/172)
eRVR	59 (210/353)	83 (288/349)	56 (97/172)
cEVR	86 (302/353)	93 (324/349)	87 (150/172)
EOTR	86 (303/353)	95 (332/349)	92 (158/172)
Virologic breakthrough	5 (16/353)	1 (2/349)	2 (3/172)
Relapse	14 (51/353)	12 (43/349)	13 (23/172)

*alfa* peginterferon alfa-2a, *cEVR* complete early virologic response, *CI* confidence interval, *DCV* daclatasvir, *diff.* difference, *EOTR* end-of-treatment response, *eRVR* extended rapid virologic response, *Lambda* peginterferon Lambda-1a, *N/A* not available, *RBV* ribavirin, *RVR* rapid virologic response, *SVR12* sustained virologic response at post-treatment Week 12, *SVR24* sustained virologic response at post-treatment Week 24

^a^Lambda-alfa difference adjusted for randomization strata. Non-inferior if lower CI bound is >−10 %; superior if both non-inferior and lower CI bound is >0 %

^b^The study was terminated before the 24 week follow-up visit of 14 patients with SVR12, hence SVR24 data for these 14 patients are unavailable

**Fig. 2:**
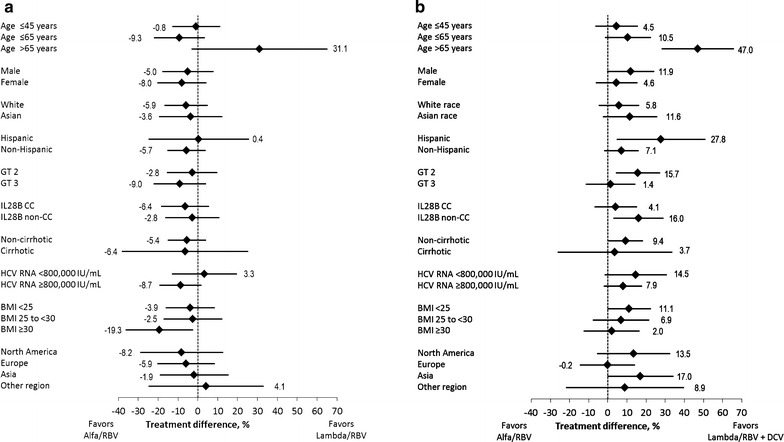
Treatment differences and 97.5 % confidence intervals by subgroup. **a** Subgroup analysis of the Lambda/RBV versus alfa/RBV treatment difference and 97.5 % confidence intervals. **b** Subgroup analysis of the Lambda/RBV + DCV versus alfa/RBV treatment difference and 97.5 % confidence intervals. *alfa* peginterferon alfa-2a, *BMI* body mass index, *DCV* daclatasvir, *GT* genotype, *Lambda* peginterferon Lambda-1a, *RBV* ribavirin

In subgroup analyses, there were no notable effects on Lambda/RBV versus alfa/RBV treatment differences with respect to gender, race (white or Asian), Hispanic/Latino ethnicity, HCV genotype, *IL28B* genotype, geographic region, or cirrhosis status (Fig. 2a). The treatment difference slightly favored alfa/RBV for patients with a body mass index ≥30 kg/m^2^ (97.5 % CI did not cross zero), although patient numbers were relatively small (Lambda/RBV, *n* = 54; alfa/RBV, *n* = 21). Treatment differences favored Lambda/RBV + DCV over alfa/RBV (97.5 % CI did not cross zero) in patients >65 years old and in Hispanic patients, although patient numbers were low for both comparisons (Fig. 2b). Lambda/RBV + DCV was also favored in patients with HCV genotype 2, patients with *IL28B* non-CC genotypes, non-cirrhotic patients, and patients in Asia (Fig. 2).

### Genotype 2

Both Lambda arms tended to have higher SVR12 rates among patients infected with genotype 2 compared with genotype 3 (Table 2). Among genotype 2-infected patients, SVR12 was achieved by 90 % in the Lambda/RBV + DCV group, 72 % in the Lambda/RBV group, and 74 % in the alfa/RBV group. One additional patient receiving Lambda/RBV + DCV had HCV RNA <LLOQ TD at week 12 and was therefore defined as a treatment failure per the study protocol; however, HCV RNA was <LLOQ TND at all post-treatment visits. The 12-week regimen of Lambda/RBV + DCV achieved non-inferiority to the 24-week regimen of alfa/RBV for SVR12 in patients infected with genotype 2, with a treatment difference of 16 % and 97.5 % CI (4.2, 27.2), whose lower limit was above −10 %. In patients with genotype 2 infection, SVR12 rates were similar in patients with or without cirrhosis: 73 % versus 71 % (Lambda/RBV), 92 % versus 89 % (Lambda/RBV + DCV), and 67 % versus 74 % (alfa/RBV). Further assessment of patients treated with Lambda/RBV + DCV, based on multivariate logistic regression analysis, found no significant effect of patient baseline factors on SVR12 rates in patients with HCV genotype 2.

### Genotype 3

Among genotype 3-infected patients, SVR12 was achieved by 75 % in the Lambda/RBV + DCV group, 64 % in the Lambda/RBV group, and 73 % in the alfa/RBV group. The 12-week regimen of Lambda/RBV + DCV did not achieve non-inferiority to the 24-week regimen of alfa/RBV for SVR12 in patients infected with genotype 3. The failure of Lambda/RBV + DCV to achieve non-inferiority for SVR12 in genotype 3 infection was driven by a higher rate of post-treatment relapse in genotype 3 (19 %) than genotype 2 (6 %). As noted earlier, SVR12 rates were higher among patients without cirrhosis in all treatment arms; this difference was driven primarily by SVR12 rates in patients with genotype 3 infection, among whom SVR12 rates for those with or without cirrhosis were 29 % versus 67 % (Lambda/RBV), 43 % versus 78 % (Lambda/RBV + DCV), and 50 % versus 75 % (alfa/RBV). In addition, there were more patients with cirrhosis among the genotype 3-infected group. Multivariate logistic regression analysis found both cirrhosis and the baseline NS5A-Y93H polymorphism to be negative predictors of SVR12 among genotype 3-infected patients when treated for 12 weeks with Lambda/RBV + DCV.

### Resistance

#### Genotype 2

Baseline NS5A sequences were available for 173/184 genotype 2-infected patients who were treated with Lambda/RBV + DCV (Additional file 1: Table S1a). Among these 173 patients, 17 were reported as not achieving SVR12. Three of these 17 non-SVR12 patients received ≤4 weeks of treatment due to death, withdrawn consent, or loss to follow-up while one patient actually achieved SVR (described above). All 17 non-SVR12 patients had baseline NS5A polymorphisms at positions F28 and/or L31. However, most patients with these common polymorphisms achieved SVR12. SVR12 was achieved by 89 % (57/64) and 91 % (99/109) of patients with or without baseline F28 polymorphisms, respectively, and by 88 % (103/117) and 95 % (53/56) of patients with or without baseline L31 polymorphisms, respectively. Thirteen genotype 2-infected patients with virologic failure had NS5A sequence data at both baseline and failure (Additional file 1: Table S2a). At failure, NS5A resistance-associated variants (RAVs) were detected in these 13 patients at positions F28 (1/13), L31 (5/13), or F28 and L31 (7/13). Treatment-emergent NS5A RAVs were present in 46 % (6/13) of patients at F28 (n = 4) or L31 (n = 2).

#### Genotype 3

Baseline NS5A sequences were available for all 165 patients with genotype 3 infection who were treated with Lambda/RBV + DCV (Additional file 1: Table S1b). SVR12 was achieved by 63 % (24/38) of patients with NS5A polymorphisms at M28, A30, L31, and/or Y93, and by 78 % (99/127) of patients without polymorphisms at any of these positions. SVR12 rates in patients with or without baseline NS5A polymorphisms at M28 or A30 were comparable. SVR12 was achieved by 50 % (2/4) and 75 % (121/161) of patients with or without M28 polymorphisms, respectively, and by 72 % (18/25) or 75 % (105/140) of patients with or without A30 polymorphisms, respectively. SVR12 rates in patients without baseline NS5A-Y93H (77 %, 119/154) were higher than in patients with this polymorphism (36 %, 4/11). Two patients had L31 polymorphisms at baseline; both achieved SVR12. NS5A sequencing data were available at both baseline and failure for 38/42 genotype 3-infected patients with virologic failure (Additional file 1: Table S2b). NS5A-Y93H was the most frequent RAV detected in this group, including 26/38 patients (68 %) with treatment-emergent Y93H and 7/38 (18 %) with Y93H at both baseline and failure. Emergent M28A, A30K, and L31F were present in 1/38, 1/38, and 1/38 patients, respectively.

#### Safety

There were no unexpected AE signals; observed events were consistent with known alfa/RBV toxicities and with previous clinical study data for Lambda/RBV (Table 3). The incidence of all-grade AEs ranged from 86 % to 97 % in the three treatment arms. Dose reductions of both interferon and RBV were less common in the two Lambda arms than in the alfa/RBV arm. Almost all dose reductions in alfa/RBV recipients were due to AEs, whereas the majority of reductions in Lambda recipients were due to elevated liver function tests.

**Table 3:** On-treatment safety

Parameter, *n* (%)	24 weeks lambda/RBV *N* = 353	12 weeks lambda/RBV + DCV *N* = 349	24 weeks alfa/RBV *N* = 172
Total with AEs (all grades)	316 (90)	299 (86)	166 (97)
Death	0	1 (<1)	0
Grade 3–4 AEs	48 (14)	24 (7)	60 (35)
SAEs	22 (6)	10 (3)	4 (2)
AEs leading to discontinuation	26 (7)	10 (3)	15 (9)
Interferon dose reductions	25 (7)	10 (3)	50 (29)
For adverse events	8 (2)	6 (2)	47 (27)
For elevated liver function tests	18 (5)	5 (1)	3 (2)
Ribavirin dose reductions	21 (6)	12 (3)	33 (19)
For adverse events	16 (5)	11 (3)	33 (19)
For elevated liver function tests	5 (1)	1 (<1)	0
Notable AEs			
Fatigue	110 (31)	87 (25)	74 (43)
Influenza-like illness	26 (7)	18 (5)	36 (21)
Asthenia	43 (12)	35 (10)	23 (13)
Pyrexia	20 (6)	23 (7)	41 (24)
Pruritus	109 (31)	82 (23)	50 (29)
Nausea	88 (25)	71 (20)	51 (30)
Insomnia	110 (31)	74 (21)	50 (29)
Headache	64 (18)	47 (13)	44 (26)
Dizziness	34 (10)	46 (13)	38 (22)
Myalgia	49 (14)	45 (13)	60 (35)
Arthralgia	48 (14)	44 (13)	49 (28)
Decreased appetite	68 (19)	51 (15)	55 (32)
Anemia	14 (4)	14 (4)	35 (20)
Neutropenia	1 (<1)	1 (<1)	53 (31)
Depression	29 (8)	16 (5)	14 (8)
Composite AEs			
Interferon-associated flu-like symptoms^a^	41 (12)	32 (9)	63 (36)
Musculoskeletal events^b^	90 (26)	72 (21)	85 (49)
Treatment-emergent grade 3–4 laboratory abnormalities			
Hemoglobin <9.0 g/L	2 (<1)	1 (<1)	8 (5)
Platelet count <5 × 10^4^/mm^3^	0	0	7 (4)
Leukocytes <1.5 × 10^3^/mm^3^	0	0	23 (13)
Lymphocytes <5 × 10^2^/mm^3^	1 (<1)	5 (1)	29 (17)
Neutrophils <7.5 × 10^2^/mm^3^	0	0	47 (27)
ALT >5.0 × ULN	22 (6)	13 (4)	5 (3)
AST >5.0 × ULN	33 (9)	12 (3)	5 (3)
Gamma-glutamyl transferase >5.0 × ULN	16 (5)	6 (2)	2 (1)
Total bilirubin >2.5 × ULN	27 (8)	9 (3)	0
Direct bilirubin >1.2 mg/dL	20 (6)	10 (3)	0
Composite treatment-emergent grade 3–4 laboratory abnormalities			
Cytopenic abnormalities^c^	10 (3)	7 (2)	63 (36)

*AE* adverse event, *alfa* peginterferon alfa-2a, *ALT* alanine aminotransferase, *AST* aspartate aminotransferase, *Lambda* peginterferon Lambda-1a, *RBV* ribavirin, *SAE* serious adverse event, *ULN* upper limit of normal

^a^Flu-like symptoms defined as pyrexia, chills, or pain

^b^Musculoskeletal events defined as myalgia, arthralgia, or back pain

^c^Cytopenic abnormalities defined as hemoglobin <10 g/dL, absolute neutrophils <750 cells/mm^3^, or platelets <50,000 cells/mm^3^

Grade 3–4 laboratory abnormalities were more common in the alfa/RBV arm, due primarily to cytopenias. Patients receiving Lambda/RBV had numerically fewer cytopenic abnormalities, flu-like and musculoskeletal events than those receiving alfa/RBV. Patients receiving Lambda/RBV + DCV had significantly fewer cytopenic abnormalities than patients receiving alfa/RBV (3 % vs 36 %; p < 0.0001), and numerically fewer flu-like and musculoskeletal events. RBV dose reduction was most common in the alfa/RBV arm (19 %) whereas the Lambda/RBV + DCV arm had the lowest rate of RBV dose reduction (3 %). Grade 3–4 anemia was observed in 5 % of the alfa/RBV group versus <1 % in the Lambda/RBV + DCV group. Grade 3–4 bilirubin elevations were observed in 8 % of the Lambda/RBV group and in 3 % of the Lambda/RBV + DCV group with no grade 3–4 elevations in the alfa/RBV group. Grade 3–4 ALT elevations were observed in 6 % of the Lambda/RBV group, 4 % of the Lambda/RBV/DCV group, and 3 % of the alfa/RBV group. Overall, the 12-week Lambda/RBV + DCV regimen appeared better tolerated than either of the 24-week arms (Table 3) with the lowest proportion of grade 3–4 AEs, discontinuations for AEs, and RBV dose reductions.

Three hepatic decompensation events were reported. A 63-year-old female with cirrhosis and a history of esophageal varices experienced hepatic decompensation during treatment with Lambda/RBV + DCV, and was discontinued from study drug on study Day 35. This patient later died of renal failure and septic shock at post-treatment Week 4. A 58-year-old male with cirrhosis experienced hepatic failure leading to discontinuation of Lambda/RBV on Day 91; the event was considered resolved on Day 115. A 56-year-old male experienced decompensated cirrhosis with elevated total bilirubin, encephalopathy, and ascites leading to discontinuation of Lambda/RBV + DCV therapy on Day 64. The bilirubin elevation improved progressively post-treatment and was considered resolved on day 162. In addition to these patients, a 40-year-old male with a history of fatty liver and Gilbert’s syndrome experienced ALT and aspartate aminotransferase (AST) increases leading to interruption of Lambda/RBV treatment on Day 56; treatment at a reduced dose resumed on Day 78. The patient completed blinded therapy but subsequent extended therapy with Lambda/RBV was discontinued on Day 146 after the events worsened to grade 4. ALT and AST levels were normal on Day 189.

## Discussion

This phase 3, randomized, double-blind study compared the efficacy and safety of Lambda/RBV, with and without DCV, with that of alfa/RBV in treatment-naive patients with genotype 2 or 3 infection. In a direct comparison of interferon/RBV treatment over 24 weeks, the endpoint of non-inferiority of Lambda/RBV versus alfa/RBV was not met for the primary endpoint of SVR12. This finding was unexpected, since phase 2 clinical data for genotypes 2 and 3 from the dose-ranging EMERGE study (Muir et al. 2014) showed a numerically higher SVR12 rate with Lambda (180 μg) plus RBV than with alfa/RBV after 24 weeks of treatment (76 vs 57 %). However, whereas the proportions of genotype 3 to genotype 2 in the Lambda and alfa arms of the EMERGE study (41–50 %) were similar to this study, patient numbers (29–30 patients per arm) were much lower.

In contrast, 12 weeks of triple therapy with Lambda/RBV and DCV was superior to 24 weeks of alfa/RBV for the primary endpoint, and also superior for early on-treatment (RVR) response. High SVR12 rates were achieved in patients with genotype 2 infection, with no significant impact of baseline demographic or disease characteristics, including cirrhosis status. However, the 12-week regimen was found to be less efficacious among patients with genotype 3, primarily due to a higher rate of relapse. This finding is similar to that noted with 12 weeks of sofosbuvir with RBV; extension of that regimen to 24 weeks resulted in a significantly higher SVR rate (Zeuzem et al. 2014). Genotype 3-infected patients with cirrhosis had substantially lower responses than those without cirrhosis; this difference was significant in multivariate analysis. The difference was larger in the Lambda arms than in the alfa/RBV arm. Treatment differences related to cirrhosis status in genotype 3 infection, together with the shorter duration of treatment with Lambda/RBV + DCV, may have contributed to the higher relapse rate for Lambda/RBV + DCV in genotype 3. Prolonging therapy for 24 weeks may potentially increase SVR12 rates in genotype 3-infected patients and reduce the impact of baseline factors; however, this has not been studied.

Baseline NS5A polymorphisms associated with DCV resistance in patients treated with Lambda/RBV + DCV had a greater impact in genotype 3 infection than in genotype 2. Most patients with genotype 2 infection had baseline polymorphisms at F28 and/or L31; however, differences in SVR12 rates in patients with or without these polymorphisms at baseline were modest. Baseline NS5A polymorphisms associated with DCV resistance were less common in patients with genotype 3 infection. However, 36 % of patients with Y93H at baseline achieved SVR12, compared with 77 % of patients without this baseline polymorphism. Moreover, Y93H was present post-failure in 86 % of genotype 3-infected patients who experienced virologic failure. Multivariate logistic regression analysis identified the presence of Y93H as having a significant adverse impact on SVR12 among genotype 3-infected patients. In follow-up studies, persistence of NS5A resistance variants was variable in patients who failed therapy with DCV-based regimens (Reddy et al. 2014). Partial or complete replacement of NS5A variants was observed in some patients after 1–2 years of post-treatment follow-up; however, the potential relevance of these findings to retreatment strategies has not yet been established.

The safety profiles of Lambda/RBV and Lambda/RBV + DCV in this study were consistent with previous studies of Lambda/RBV (Muir et al. 2014; Vierling et al. 2013). The lower incidences of hematologic, musculoskeletal, and flu-like symptoms and lower rates of dose adjustment for Lambda-based regimens versus alfa-2a that were observed are consistent with the more restricted extrahepatic distribution of the Type III receptor and its virtual absence from bone marrow progenitors and peripheral blood cells (Andersen et al. 2013). There were no unexpected safety findings. The proportions of grade 3–4 aminotransferase and bilirubin elevations over 24 weeks on Lambda/RBV in this study were similar to those seen over 24 weeks in the EMERGE genotype 2 and 3 population among patients receiving 120 or 180 μg of Lambda. Similarly, the proportions of grade 3–4 aminotransferase and bilirubin elevations over 12 weeks of Lambda/RBV + DCV (≈3.5 and ≈3 %, respectively) were comparable to those observed with the same regimen over 24 weeks in the phase 2 D-LITE study (5 and 3 %, respectively; Muir et al. 2014; Vierling et al. 2013). Hepatic decompensation occurred in 3 patients, all with prior evidence of portal hypertension. Similar events have been reported in previous studies using alfa interferons (Hezode et al. 2013).

In conclusion, the results of this large multinational phase 3 study in treatment-naive HCV genotype 2 and 3 infection demonstrated that 12 weeks of treatment with Lambda/RBV and DCV gave a superior SVR12 rate compared with 24 weeks of alfa/RBV in the combined population of patients with genotype 2 or 3 infection. However, the regimen was less effective among genotype 3-infected patients with cirrhosis. The shorter, Lambda-containing regimen also demonstrated better tolerability than alfa/RBV and a lower incidence of interferon and/or RBV dose adjustments, consistent with phase 2 clinical data for Lambda/RBV with and without DCV. Despite the encouraging efficacy and safety outcomes of the Lambda/RBV + DCV regimen in this study, further development of this regimen was discontinued due to the emergence of highly efficacious and well-tolerated all-oral DAA combination regimens. However, data from the current study remain relevant in the context of understanding the clinical profile of DCV, which has been widely approved as a component of all-oral HCV regimens.

## Additional file

10.1186/s40064-016-2920-z Baseline NS5A polymorphisms at 28, 30, 31, and/or 93 in HCV genotype 2-infected (Table 1a) and genotype 3-infected (Table 1b) patients who received DCV-containing regimen. **Table S2.** Newly emergent NS5A substitutions in virologic failures with HCV genotype 2 (Table 2a) or genotype 3 (Table 2b).

## Abbreviations

HCV: hepatitis C virus
Alfa: peginterferon alfa-2a
RBV: ribavirin
AE: adverse event
SVR: sustained virologic response
DAA: direct-acting antiviral
Lambda: peginterferon lambda-1a
DCV: daclatasvir
ULN: upper limit of normal
ALT: alanine aminotransferase
SVR12: sustained virologic response at post-treatment Week 12
LLOQ: lower limit of quantitation
RVR: rapid virologic response
eRVR: extended rapid virologic response
cEVR: complete early virologic response
EOTR: end-of-treatment response
mITT: modified intention-to-treat
CI: confidence interval
RAV: resistance-associated variant
AST: aspartate aminotransferase
