# Supplementary material for: A randomized, controlled study of peginterferon lambda-1a/ribavirin ± daclatasvir for hepatitis C virus genotype 2 or 3
Source: Springerplus. 2016 Aug 19;5(1):1365. doi: 10.1186/s40064-016-2920-z (PMC4990525; doi:10.1186/s40064-016-2920-z)
Supplement: Supplementary file 1 — 10.1186/s40064-016-2920-z Baseline NS5A polymorphisms at 28, 30, 31, and/or 93 in HCV genotype 2-infected (Table 1a) and genotype 3-infected (Table 1b) patients who received DCV-containing regimen. Table S2. Newly emergent NS5A substitutions in virologic failures with HCV genotype 2 (Table 2a) or genotype 3 (Table 2b). [file 40064_2016_2920_MOESM1_ESM.pdf]

Supplementary Table 1. Baseline NS5A polymorphisms at 28, 30, 31, and/or 93 in HCV genotype 2-infected (Table 1a) and genotype 3-infected (Table 1b) patients who received DCV-containing regimen

Table 1a.

	Genotype 2		
**Parameter**	**Total**	**SVR12**	**Non-SVR12**
Total patients	184	165	19
With BL NS5A sequence [a]	173	156	17 [b]
**NS5A polymorphisms at: n/N (%)**			
F28 or K30 or L31 or Y93	156/173 (90)	139/156 (89)	17/17 (100)
F28C, L	64/173 (37)	57/156 (37)	7/17 (41)
K30R	6/173 (3)	6/156 (4)	0
L31M, V [c]	117/173 (68)	103/156 (66)	14/17 (82)
Y93	0	0	0

Table 1b.

	Genotype 3		
**Parameter**	**Total**	**SVR12**	**Non-SVR12**
Total patients	165	123	42 [d]
With BL NS5A sequence	165	123	42
**NS5A polymorphisms at: n/N (%)**			
M28 or A30 or L31 or Y93	38/165 (23)	24/123 (20)	14/42 (33)
M28L, V	4/165 (2)	2/123 (2)	2/42 (5)
A30K, S, T	25/165 (15)	18/123 (15)	7/42 (17)
L31M	2/165 (1)	2/123 (2)	0
Y93H	11/165 (7)	4/123 (3)	7/42 (17)

[a] Nine of the 11 patients without available baseline sequences achieved SVR12. One of the 2 patients without SVR12 had a single HCV RNA value >LLOQ TD at the end of treatment (defined as treatment failure by the study protocol) but HCV RNA was <LLOQ TND at subsequent post-treatment visits. The other patient without SVR12 withdrew from the study after 1 week of treatment.

[b] Among the 17 patients without SVR12, one patient died at post-treatment Week 4, two patients withdrew consent and were lost to subsequent follow-up, and one patient achieved SVR12 but had a single measurement of HCV RNA >LLOQ at post-treatment Week 24, followed by <LLOQ TND at post-treatment Week 36.

[c] One patient had a mixture of L31I/M; 2 patients had mixtures of L31M/V while all other patients had L31M.

[d] Among the 42 patients without SVR12, 2 patients discontinued study therapy at 2 weeks of treatment with either HCV RNA <LLOQ TD or <LLOQ TND at the last visit before being lost to follow up.

BL, baseline; SVR12, sustained virologic response at post-treatment Week 12.

Supplementary Table 2. Newly emergent NS5A substitutions in virologic failures with HCV genotype 2 (Table 2a) or genotype 3 (Table 2b)

Table 2a

**Parameter**	**Genotype 2**
Non-responder, *n*	19
With NS5A sequence post- baseline, *n*	13
With baseline and post-baseline NS5A sequence, *n*	13
With emergent F28, K30, L31, or Y93 variants, *n/N* (%)	6/13 (46)
Noted emergent NS5A RAVs, *n/N* (%)	
F28C, G, S	4/13 (31)
K30	0
L31M	2/13 (15)
Y93	0
C92S	1/13 (8)

Table 2b

**Parameter**	**Genotype 3**
Non-responder, *n*	42
With NS5A sequence post-baseline, *n*	38
With baseline and post-baseline NS5A sequence, *n*	38
With emergent M28, K30, L31, or Y93 variants, *n/N* (%)	28/38 (74)
Noted emergent NS5A RAVs, *n/N* (%)	
M28L, A	2/38 (5)
A30K	1/38 (3)
L31F, V	1/38 (3)
Y93H	26/38 (68)

RAV, resistance-associated variant
